# Analysis of cellular and cell free mitochondrial DNA content and reactive oxygen species levels in maternal blood during normal pregnancy: a pilot study

**DOI:** 10.1186/s12884-022-05156-2

**Published:** 2022-11-16

**Authors:** Anubhav Srivastava, Pransu Srivastava, Shashank Mathur, Suman Mishra, Sabiya Abbas, Amrit Gupta, Prabhaker Mishra, Meenakshi Tiwari, Lokendra Kumar Sharma

**Affiliations:** 1 Department of Molecular Medicine & Biotechnology, Sanjay Gandhi Post Graduate Institute of Medical Sciences, 4th Floor, PMSSY Building, Raebareli Road, Lucknow, Uttar Pradesh 226014 India; 2 Department of Maternal & Reproductive Health, Sanjay Gandhi Post Graduate Institute of Medical Sciences, Lucknow, 226014 India; 3 Department of Biostatistics and Health Informatics, Sanjay Gandhi Post Graduate Institute of Medical Sciences, Lucknow, 226014 India; 4 Department of Pathology and Laboratory Medicine, All India Institute of Medical Sciences, Patna, 801507 India

**Keywords:** Pregnancy, Mitochondrial DNA, Reactive oxygen species, Oxidative stress

## Abstract

**Background:**

Alterations in mitochondrial signatures such as mitochondrial DNA (mtDNA) content in maternal blood have been linked to pregnancy-related complications. However, changes in maternal mtDNA content, their distribution and associated signaling during normal pregnancies are not clear; which could suggest their physiological role in maternal adaptation to pregnancy related changes and a reference threshold. The aim of this study: to assess the distribution of mtDNA in peripheral blood and their association with circulatory ROS levels across different trimesters of healthy pregnancy.

**Methods:**

In this pilot cross sectional study, blood samples of normal pregnant women from each trimester (total = 60) and age-matched non-pregnant (NP) women as control group (*n* = 20) were analyzed for a) the relative distribution of mtDNA content in cellular and cell free (plasma) fractions using relative quantitative polymerase chain reaction (qPCR) and b) the levels of circulating reactive oxygen species (ROS) by measurement of plasma H_2_O_2_. The results were compared between pregnant and NP groups and within trimesters for significant differences, and were also analyzed for their correlation between groups using statistical methods.

**Results:**

While, we observed a significant decline in cellular mtDNA; plasma mtDNA was significant increased across all trimesters compared to NP. However, from comparisons within trimesters; only cellular mtDNA content in 3rd trimester was significantly reduced compared to 1st trimester, and plasma mtDNA did not differ significantly among different trimesters. A significantly higher level of plasma H_2_O_2_ was also observed during 3rd trimester compared to NP and to 1st trimester. Correlation analysis showed that, while cellular mtDNA content was negatively correlated to plasma mtDNA and to plasma H_2_O_2_ levels; plasma mtDNA was positively correlated with plasma H_2_O_2_ content.

**Conclusions:**

This study suggested that normal pregnancy is associated with an opposing trend of reduced cellular mtDNA with increased circulatory mtDNA and H_2_O_2_ levels, which may contribute to maternal adaptation, required during different stages of pregnancy. Estimation of mtDNA distribution and ROS level in maternal blood could show mitochondrial functionality during normal pregnancy, and could be exploited to identify their prognostic/ diagnostic potential in pregnancy complications.

**Supplementary Information:**

The online version contains supplementary material available at 10.1186/s12884-022-05156-2.

## Background

Mitochondria play a central role in multiple functions such as cellular growth and apoptosis through bioenergetics and metabolism [1]. Therefore, mitochondrial defects and oxidative stress have been linked to pregnancy related complications leading to developmental delay and other neonatal disorders [2]. Pregnancy is a systemic process of development of fetus; driven by cell growth, migration and differentiation process leading to tissues and organ development [3]. Several studies have shown that mitochondria play an important role in the maintenance of pregnancy and neonatal development through regulation of energy, metabolism, hormone synthesis and oxygen sensing capacity [4–6]. Therefore, mitochondrial signatures including mtDNA content and free radicals in circulation have now been investigated for their potential to show mitochondrial functionality, oxidative stress and link to pregnancy complications [7]. Mitochondria contain its own genetic material; mitochondrial DNA (mtDNA), which is approximately 16.5 kb in size and transmitted by the maternal lineage. Somatic cells contain hundreds to thousands of mitochondria, each having 2 to 10 copies of mtDNA per mitochondrion [8]. However, it varies in post-mitotic tissues, which highly depend on energy and oxidative metabolism, and mitochondria are present in high numbers with higher mtDNA content. Similarly, a physiologic state with high-energy demand, such as fertilization and pregnancy, also requires greater mitochondrial activity [9]. A higher mitochondrial number with increased volume of ooplasm has been linked to a high rate of fertilization [10]. Since mitochondria are the major source of reactive oxygen species (ROS), mitochondrial dysfunction and oxidative stress have been observed in pregnancy complications [11]. Altered mtDNA content has been reported in pre-eclampsia (PE), intrauterine growth restriction (IUGR) and placental disruption cases, showing mitochondrial stress during pregnancy complications [12–14]. Compared to pregnancy complications, limited information is available regarding the regulation of mtDNA content during normal pregnancies. Very few studies have reported progressive changes in mtDNA content from whole blood or serum during normal pregnancy [13, 15]. However, the opposing patterns of mtDNA change from these studies also raise a possibility that mtDNA could be differentially distributed in cells or circulation and their relative distribution could show the mitochondrial response during physiological pregnancy. A comparative investigation on mtDNA content in blood cells and circulation and associated signaling during normal pregnancy is largely lacking, which could indicate mitochondrial contribution in maternal adaptation during physiological changes in pregnancy. In this study, we partly addressed this issue by investigating the relative distribution of mtDNA content in cellular and plasma fractions of blood from different trimesters of healthy pregnancy and compared with age-matched non-pregnant females. Since mitochondria are the major source of free radical generation, association with free radical signaling in different trimesters of healthy pregnancy was investigated through measuring plasma H_2_O_2_ levels and their correlation with mtDNA content.

## Methods

### Subject information

This is a pilot cross sectional study on normal pregnant women and age-matched non-pregnant (NP) females as controls who visited the department of maternal and reproductive health (MRH) of Sanjay Gandhi Post Graduate Institute of Medical Sciences (SGPGIMS); a tertiary care center in north India. Subjects visited the center for routine antenatal care during their pregnancy or routine gynecological issues and screened to take part in this study through written informed consent. The institutional ethics committee approved the study (IEC code: 2019–86-IP-109, Ref. no: PGI/BE/492). The study population comprised total 80 participants, including 20 each from different trimesters: 1st (0-13th week), 2nd (14th–26th week) and 3rd (27th week-till delivery) as pregnancy group along with 20 NP females from reproductive age group as control group. Subjects were included in the study based on screening of medical history along with physical examination. Exclusion criteria for pregnancy group included an overt history of chronic disease, gestational hypertension or preeclampsia, twin pregnancies, gestational diabetes, use of recreational drugs or hormonal contraceptives within the previous 6 months, use of fertility treatments or supplements, and menstrual irregularities and smoking. Inclusion criteria included all those pregnant females who were in their 1st /2nd/3rd trimester and above 18 years of age. For the control group, non-pregnant females of reproductive age group with normal menstrual cycles and no previous history of chronic illness, no gynecological problems and not on any medication were included. As applicable, the variables such as age, blood pressure (BP), body mass index (BMI), gestational age (GA) were recorded at the time of enrolment, and followed for measuring BP and GA weeks during their visits and baby weight at the time of delivery. In pregnant women, GA was estimated based on the first day of their last menstrual period and confirmed by trans-vaginal ultrasound at the time of recruitment. Routine hematological investigations in pregnancy subjects such as hemoglobin, hematocrit, total leukocyte counts, and differential leukocyte (monocytes, lymphocytes, eosinophils, neutrophils, and platelets) were done in clinical laboratory using automated hematology analyzer and data was collected retrospectively. All women included in this study were free of apparent infections within the uterus or amniotic cavity. Routine ultrasound scans and normal intrauterine fetal growth (as and when required) were performed to ensure healthy pregnancy in the pregnancy group.

### Sample collection

Peripheral venous blood sample (5 ml) from the subjects was collected via an intravenous catheter inserted into the antecubital vein of the left arm and collected in BD Vacutainer® spray-coated K2EDTA Tubes (BD Inc.). The samples were immediately processed for blood cells and plasma separation by centrifugation at 3000 rpm at 4 °C for 10 min, which provided plasma in the supernatant and the lower part with RBCs and buffy coat enriched in WBCs and platelets. Plasma was carefully removed using pasture pipette to avoid mixing with the buffy coat. The buffy coat and RBCs were mixed gently for cellular fraction. Aliquots of these plasma and cellular fractions were processed for DNA isolation on the same day. Remaining samples were stored in aliquots at − 80 °C until further processing.

### DNA isolation and quantitative PCR (qPCR)

Total DNA from 250 μl from cellular or plasma samples was isolated by QIAamp DNA isolation Kit as per the manufacturer protocol (Qiagen India Pvt. Ltd). Quantification of DNA was done using Nanodrop ND2000 UV-visible spectrophotometer (Thermo-Fisher Scientific Inc.) at A_260_nm and quality was assessed by A_260_/A_280_ ratio (ranged from 1.8–2). An amount of 20 ng of total DNA was used for mt-DNA copy number determination through relative amplification of mt-DNA or nuclear DNA in separate reactions using qPCR. Mitochondrial ND1 (mtND1) gene primers (fwd: 5′-CACCCAAGAACAGGGTTTGT-3′ and rev: 5′-TGGCCATGGGTATGTTGTTAA-3′) and nuclear 18 s r-RNA gene primers (fwd: 5′-TAGAGGGACAAGTGGCGTTC-3′ and rev-5′-CGCTGAGCCAGTCAGTGT-3′) were used. Nuclear 18 s RNA gene was used as an internal reference gene for normalizing mtND1 gene amplification in each case. TB green based relative amplification was performed using qPCR following manufacturer’s instructions (Takara Bio.) and run on 7900HT Fast Real-Time PCR System (Applied Biosystem). The qPCR conditions were as follows: initial denaturation for 3 min at 95 °C; followed by 40 cycles at 95 °C for 15 sec; and 60 °C for 60 sec. Relative expression of mtDNA verses nDNA was calculated by 2^−ΔCt^ (ΔC_t_ = C_t_ mtDNA - C_t_ nDNA), following previously published method [16], and fold changes were calculated compared to NP as control group.

### Reactive oxygen species measurement

H_2_O_2_ is the most common ROS and therefore H_2_O_2_ levels were estimated in the plasma samples by H_2_O_2_ content assay kit according to the manufacturer’s instruction (Immunotag, USA). The kit is based on the principle that H_2_O_2_ reacts with titanium sulfate and generates yellow titanium peroxide complex with the characteristic absorption at 415 nm. Briefly, 100 μl of plasma samples were de-proteinated with reagent I (0.9 ml) and centrifuged at 8000 g at 4 °C for 10 minutes. The supernatant was collected and mixed with reagent 2 and 3, centrifuged at 4000 g at room temperature for 10 minutes. Resulting pellet was dissolved in reagent 4 and absorbance was taken at 415 nm. The absorbance of samples was normalized with reference control and calculated as μmole per ml from standard graph. The final values were presented as relative fold change to NP.

### Statistical analysis

Kolmogorov Simonov test (KS test) was used to test the normal distribution of the continuous variables. One way ANOVA test/ Kruskal–Wallis H-test was used to compare the means/median respectively, between the four groups (1st, 2nd, 3rd trimester and NP). Continuous normally distributed variables are presented in mean ± standard deviation (SD) and for non-normal variables the median with interquartile range (IQR) are included. When result was significant at *p* < 0.05, multiple comparison using Bonferroni correction was used. To test the linear relationship between the continuous variables [among different groups for mtDNA content (cellular vs plasma) vs plasma H_2_O_2_ levels], Pearson correlation coefficient was used. The Cohen effect size (Cohen’s d) [using formula: mean difference / pooled standard deviation], was calculated to present the actual change in cellular mtDNA, plasma mtDNA and plasma H_2_O_2_ between the four groups (1st, 2nd, 3rd trimester and NP). The comparison graphs were prepared using GraphPad version 9 and statistical analysis was done using Statistical package for social sciences, version-23 (SPSS-23, IBM, Chicago, USA). A *p* value < 0.05 was considered as statistically significant.

## Results

In this study, age matched non-pregnant females: NP (*n* = 20) and healthy pregnant females (*n* = 60), 20 from each trimester were selected following institutional ethical guidelines.

As listed in Table 1, the participant baseline characteristics such as age, BP and BMI of pregnant group did not differ significantly compared to NP group. Among different trimesters; except GA, all other parameters such as BP at delivery, weeks at delivery and baby weight showed no significant variations in pregnant group. Similarly, from hematological analyses of blood samples, only platelet counts were significantly lower in 3rd trimester (*p* = 0.003) compared to 1st trimester participants, while other blood analysis result did not differ significantly among different trimesters. Changes in mtDNA content in the total DNA isolated from cellular and plasma fractions from these participants were analyzed. In addition, plasma H_2_O_2_ level was also measured from these subjects.

**Table 1:** Clinical characteristics of the study population

Characteristics	Non-Pregnant (NP) (***n*** = 20)	1st trimester (***n*** = 20)	2nd trimester (***n*** = 20)	3rd trimester (***n*** = 20)	***P*** Value*
**At enrolment**					
Age (Years)	27.69 ± 4.85	28.11 ± 3.87	28.5 ± 3.51	31.41 ± 6.38	0.112
BMI (Kg/m^3^)	23.41 ± 2.50	25.52 ± 3.65	24.88 ± 3.93	24.25 ± 3.69	0.764
SBP/DBP (mmHg)	117.72 ± 9.80/ 78.36 ± 8.22	120.76 ± 11.17/ 75.53 ± 10.29	117.26 ± 11.62/ 70.53 ± 9.92	115.31 ± 10.88/ 71.21 ± 10.15	0.592/ 0.142
Gestational Age (weeks)	N/A	7.72 ± 1.90	16 ± 2.67	32.8 ± 1.30	0.000^#^
**Haematological changes during pregnancy**					
Haemoglobin (g/dL)		10.83 2.14	11.81 ± 0.83	11.7 ± 1.19	0.128
Hematocrit (%)		35.56 ± 5.20	37.96 ± 2.35	38.22 ± 5.20	0.258
TLC (×10^9^/L)		9.77 ± 2.62	8.97 ± 2.36	10.39 ± 2.69	0.317
Monocytes (%)		3.30 ± 1.37	2.53 ± 0.99	3.26 ± 1.33	0.178
Lymphocytes (%)		23.30 ± 5.79	21.86 ± 7.44	23.8 ± 12.61	0.839
Eosinophils (%)^a^		2.75 ± 2.45 [2 (1.3,3)]	2.13 ± 1.18 [2(1,3)]	2.93 ± 1.57 [2(2,3)]	0.440
Neutrophils (%)		70.84 ± 6.01	73 ± 7.79	70 ± 13.42	0.690
Platelets (×1000/mm^3^)		258.07 ± 108.63	182.2 ± 57.10	157.43 ± 56.23	0.003^
**At delivery**					
SBP/DBP (mmHg) at delivery		121.1 ± 5.56/ 79.6 ± 5.77	120.8 ± 7.33/ 79.33 ± 7.88	118.36 ± 9.34/ 79.63 ± 8.93	0.557/ 0.994
Gestational Age (weeks) at Delivery		37.31 ± 0.80	37.12 ± 0.79	36.94 ± 1.87	0.809
Baby weight (kg)		2.66 ± 0.84	2.77 ± 0.54	2.76 ± 0.39	0.920

Data are presented in mean ± standard deviation (SD). One-way ANOVA test used to compare the means among four groups followed by multiple comparisons (Bonferroni correction)

*P-value represents post ANOVA significance between groups and within groups

# Multiple comparisons of GA in trimesters: Between 1 and 2 (*p* < 0.001), 1–3 (*p* < 0.001) and 2–3 (*p* < 0.001)

^ Multiple comparisons of platelets in trimesters:: Between 1 and 2 (*p* = 0.103), 1–3(*p* = 0.003), 2–3 (*p* = 0.542)

^a^Data are also presented as median interquartile range (IQR) within parenthesis compared by Kruskal Wallis H test

### Reduced mt-DNA content in peripheral blood cells during pregnancy

**Fig. 1:**
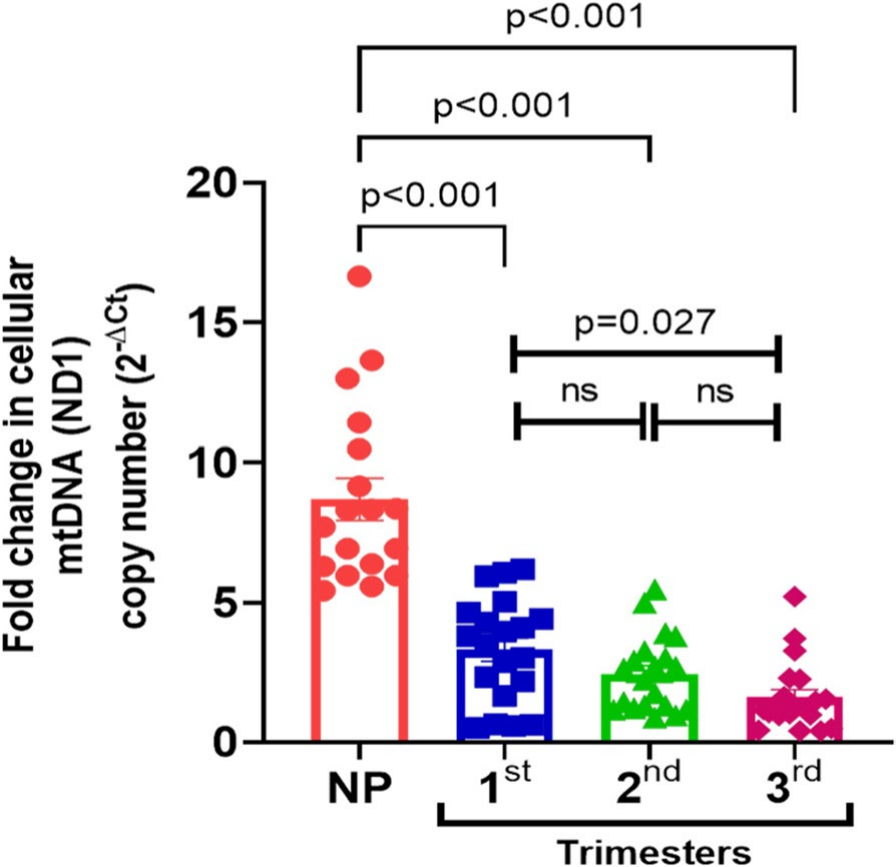
Relative quantification of mtDNA content in cellular fraction: Total DNA from cellular fraction of blood from non-pregnant (NP) and from pregnant women of different trimesters (1st, 2nd and 3rd) was used for quantitative PCR. Amplification of mtDNA gene (ND1) was normalized to 18sRNA nuclear gene amplification, using 2^-ΔCt^ method, and presented as relative to NP as control. Significance (*P* value as indicated) from NP and within different trimester was calculated by Kruskal–Wallis H-test and post-hoc analysis by Bonferroni Correction. ns: non-significant

Analysis of mtDNA content was performed by relative quantitation based on qPCR by amplifying mtDNA (mtND1 gene) and normalizing to nuclear reference 18S rRNA gene amplification (Fig. 1). Relative fold changes in mtDNA content in different trimesters (1st/2nd/3rd trimester) of pregnancy were calculated using 2^-ΔCt^ method and compared to NP females as control. As shown in Fig. 1, mtDNA content was decreased in cellular fraction of blood throughout pregnancy compared to NP. Compared to NP as 100% [mean ± SD: 8.70 ± 3.17, median (IQR): 8.01 (6.21, 10.72)] mt-DNA content was significantly reduced;-to 38.14% in 1st trimester [mean ± SD: 3.32 ± 1.86, median (IQR): 3.65 (1.78, 4.57), *p* < 0.001]; −to 28.10% in 2nd trimester [mean ± SD: 2.44 ± 1.34, median (IQR): 2.44 (1.24, 3.20) *p* < 0.001]; and -to 18.44% in 3rd trimester [mean ± SD: 1.60 ± 1.22, median (IQR): 1.21 (0.97, 2.11) *p* < 0.001]. While comparison among different trimesters; a significant reduction (48.34%) was observed in 3rd trimester only (*p* = 0.027), compared to 1st trimester and did not differ significantly between 1st vs 2nd and 2nd vs 3rd trimesters. However, normalization of mtDNA copy number with their respective platelet count in different trimesters resulted in no significant differences among different trimesters including the difference between 3rd vs 1st trimester (supplementary Fig. 1). Overall, relative quantitation of mtDNA copy number in cellular fraction suggested a progressive and significant decline in mtDNA content in all three trimesters during pregnancy compared to NP group. However, within different trimesters, cellular mtDNA content was significantly reduced only in the late gestation (3rd trimester), possibly because of reduced platelet counts compared to early gestation period (1st trimester).

### Increased circulating mt-DNA content during pregnancy

**Fig. 2:**
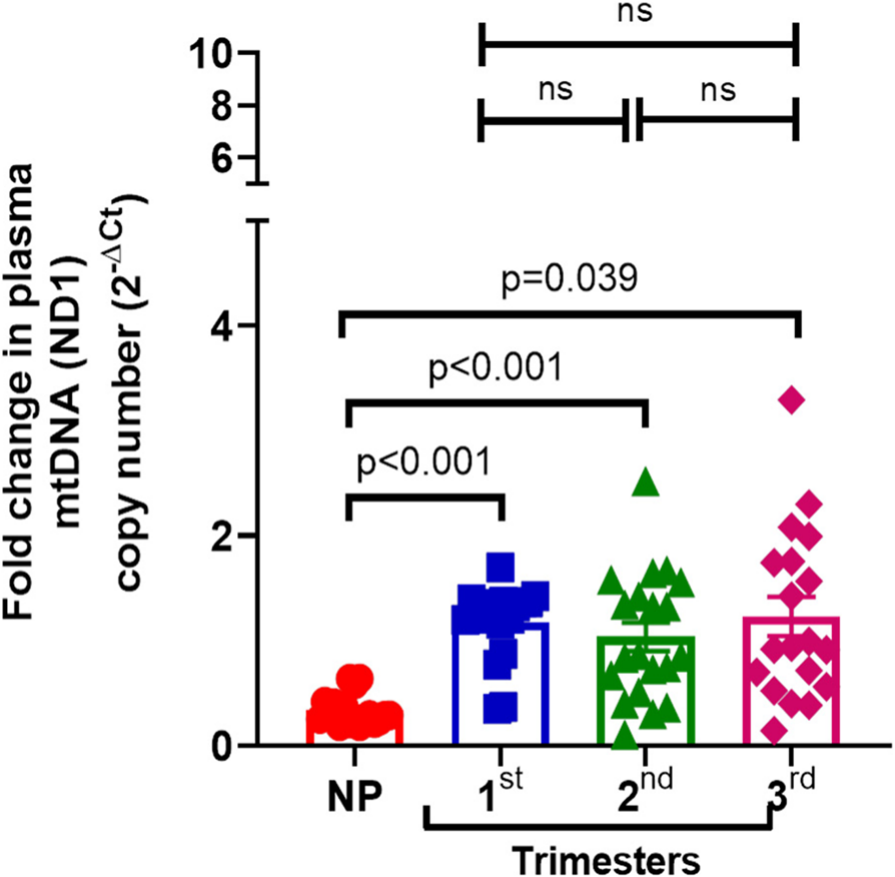
Relative quantification of mt-DNA content in plasma fraction: Total DNA from plasma of non-pregnant (NP) and pregnant women of different trimesters (1st, 2nd and 3rd) was used for quantitative PCR using similar methods as described earlier. Significance (*P* value, as indicated) from NP and within different trimester was calculated by Kruskal–Wallis H-test and post hoc analysis by Bonferroni Correction. ns: non-significant

The level of mtDNA content in circulation was determined in the total DNA isolated from plasma samples using similar relative quantitation method as mentioned earlier. Compared to the NP group, a significant increase was observed in plasma mtDNA content throughout all trimesters (Fig. 2). As shown in Fig. 2, compared to NP control [mean ± SD: 0.34 ± 0.14, median (IQR): 0.29 (0.23, 0.41], mtND1 copy number was significantly increased to 3.45-fold in 1st trimester [mean ± SD: 1.17 ± 0.35, median (IQR): 1.30(1.09, 1.37), *p* < 0.001]; 3.05-fold in 2nd trimester [mean ± SD: 1.04 ± 0.60, median (IQR): 0.85(0.55, 1.52), *p* < 0.001] and 4.74-fold in 3rd trimester [mean ± SD: 1.61 ± 1.87, median (IQR): 0.97(0.60, 1.93), *p* = 0.039]. However, no significant difference was observed when compared among different trimesters. Altogether circulating mtDNA content was significantly elevated during pregnancy than non-pregnant condition, but did not change significantly within different trimesters of pregnancy.

### Increased circulatory H_2_O_2_ levels during pregnancy

**Fig. 3:**
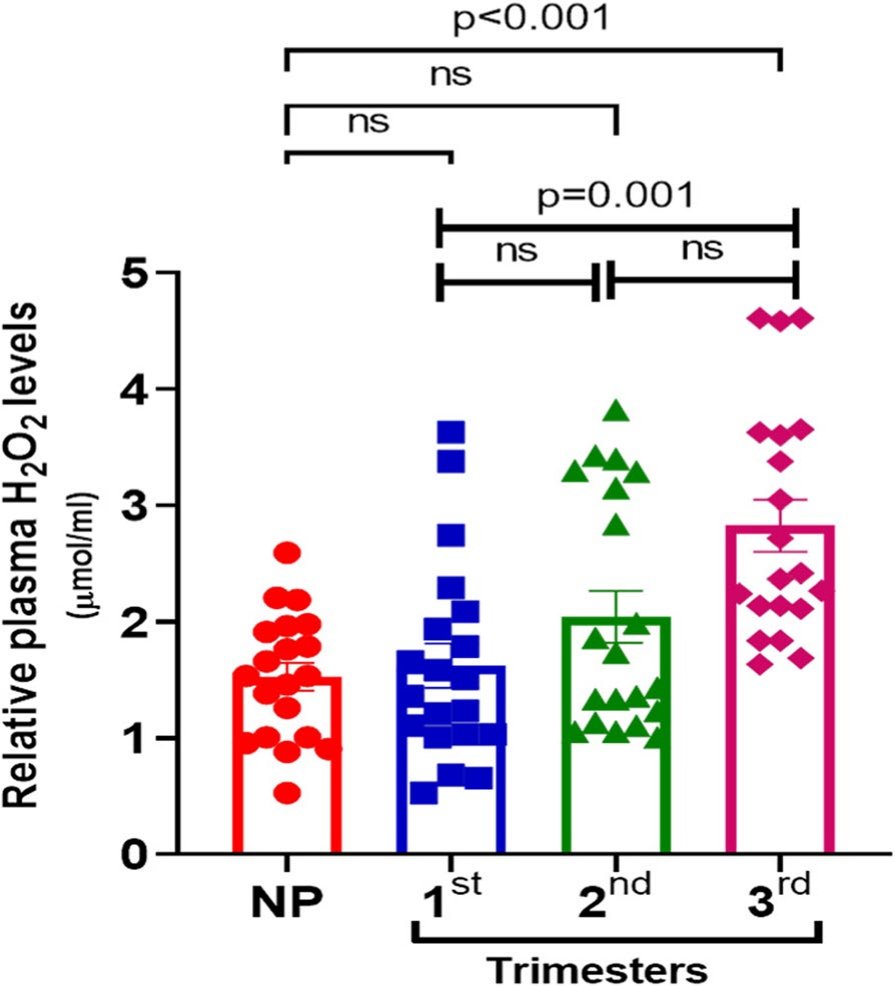
Measurement of plasma H_2_O_2_ content: Equal volume of plasma was used for measuring H_2_O_2_ level using manufacturer’s instructions and presented as relative to NP as control. Significance (*P* values as indicated) from NP and within different trimester was analyzed by One way ANOVA (data was normally distributed) and post-hoc analysis by Bonferroni correction for multiple comparison. ns = non-significant

Circulating level of H_2_O_2_ was measured in plasma samples of NP, and pregnant women in different trimester using colorimetry based H_2_O_2_ content assay kits following protocol as described in materials and methods. The circulating plasma H_2_O_2_ levels in different trimesters were compared relative to NP and within different trimesters (Fig. 3). Compared to the NP control (mean ± SD: 1.53 ± 0.53), a significant 1.85 fold increase was observed in 3rd trimester (mean ± SD: 2.82 ± 0.99, *p* < 0.001) only. In addition, within different trimesters, the increase in 3rd trimester was significant (1.74 fold, *p* = 0.001) only in comparison to 1st trimester (mean ± SD 1.62 ± 0.855). Altogether, a significant increase in H_2_O_2_ level was observed in maternal plasma in late gestation period (3rd trimester) compared to 1st trimester or non-pregnant group.

### Size effect analysis

To compare the absolute differences in the mean values of variables (mtDNA and H_2_O_2_) among these four groups, Cohen effect size (Cohen’s d) was calculated. The effect size between 0.2–0.49 was considered as small, while 0.5–0.79 and ≥ 0.8 were considered as moderate and large effect size respectively. As shown in Table 2, the higher difference was observed between value of the NP to the value of the 1st, 2nd and 3rd trimesters for cellular mtDNA (ranging from 2.06–2.94) as well as for plasma mtDNA (ranging from 0.95–3.05). Similarly, within different trimesters, the higher difference was observed only in 1st vs 3rd trimester (1.08) for cellular mtDNA. However, for plasma H_2_O_2_, higher differences were recorded only between NP vs 3rd and 1st vs 3rd trimester (1.62 and 1.29 respectively). Altogether, it suggested that the observed significant differences in cellular/plasma mtDNA in pregnant group (in all three trimesters) compared to NP group was due to larger size effect. However, for plasma H_2_O_2_ the larger size effect within trimesters was observed only in 3rd trimester compared to NP or 1st trimester.

**Table 2:** Cohen’s Effect Size (Cohen’s d^*^) of the mean difference between different groups

Groups→ Variable↓	NP vs 1st Trimester	NP vs 2nd Trimester	NP vs 3rd Trimester	1st vs 2nd Trimester	1st vs 3rd Trimester	2nd vs 3rd Trimester
**Cellular mtDNA**	2.068024	2.565929	2.948257	0.537623	1.085961	0.651487
**Plasma mtDNA**	3.054079	1.590088	0.955076	0.272926	0.324564	0.411512
**Plasma H**_**2**_**O**_**2**_	0.135034	0.641112	1.622518	0.450437	1.294916	0.780974

^*^Cohen’s d was determined by calculating the mean difference between indicated groups, and then dividing the result by the pooled SD using the formula Cohen’s d = (M2 - M1) / SD_pooled_, where SD_pooled_ = √((SD_1_^2^ + SD_2_^2^) / 2). Criteria for size effect: 0.2–0.49 = small, 0.5–0.79 = medium and ≥ 0.8 = large size effect. Higher values indicate higher difference as underlined

### Correlation of mtDNA levels with H_2_O_2_ levels

To measure the overall correlation of changes in mtDNA content between cellular vs plasma fraction, or mtDNA content to plasma H_2_O_2_ levels, Pearson’s correlation analysis was performed. As summarized in Table 3, among all groups together; cellular mtDNA was negatively correlated with plasma mtDNA content (Pearson’s *r* = − 0.350, *p* = 0.002) as well as to plasma H_2_O_2_ levels (Pearson’s *r* = − 0.301, *p* = 0.007). Contrastingly, plasma mtDNA content was positively correlated with plasma H_2_O_2_ content (Pearson’s *r* = 0.255, *p* = 0.026). However, there was no significant correlation when analyzed among NP vs 1st/2nd/3rd or within trimesters (data not shown in table). Overall, from all groups together a negative correlation between cellular mtDNA with plasma mtDNA/H_2_O_2_, and a positive correlation between plasma mtDNA with plasma H_2_O_2_ levels were detected.

**Table 3:** Correlation between cellular, plasma mtDNA and H_2_O_2_ levels (*N* = 80)

Correlation between the Variables	Pearson Correlation	***P*** value
Cellular mtDNA & Plasma mtDNA	−0.350	**0.002**
Cellular mtDNA & Plasma H_2_O_2_	−0.301	**0.007**
Plasma mtDNA and Plasma H_2_O_2_	0.255	**0.026**

Pearson correlation coefficient was calculated using SPSS. *P* < 0.05 significant (at 2-tailed)

## Discussion

Present study reports the trend of 1) mtDNA at cellular level and cell-free state, and 2) circulating ROS levels in the blood of normal pregnant females in comparison with non- pregnant females as controls. To the best of our knowledge, this is the first study to simultaneously analyze the mtDNA content in cellular and plasma fractions of blood along with plasma H_2_O_2_ levels in different trimesters of pregnancy and their comparison with non-pregnant group. Our results show that during normal pregnancy, the maternal mtDNA content displays an opposing trend in cellular vs plasma factions, compared to the non-pregnant group. We found that cellular mtDNA content declines significantly and circulating plasma mtDNA content increases significantly; in all three trimesters compared to non-pregnant conditions. However from the comparisons across different trimesters in pregnant group, the decrease in cellular mtDNA was significant only in 3rd trimester compared to 1st trimester and no significant difference was observed for plasma mtDNA content in different trimesters. Our results are in accordance with previous independent reports on the analyses of mtDNA content in whole blood or in circulation [13, 15]. For example, in a cross-sectional study on analyzing mtDNA content in normal pregnancy, Colleoni et al. reported a progressive decline in the mtDNA content in the whole blood from different trimesters, compared to non-pregnant women; and the maximum decline in mtDNA was observed in 3rd trimester compared to 1st and 2nd trimester [13]. In our study, we used cellular and plasma fractions from whole blood, and our cellular mtDNA analysis result suggested a significant decline in mt-DNA content in all three trimester compared to non-pregnant; similar to the previous report by Colleoni et al. [13]. However, while comparing among different trimesters; the reduction in mtDNA content was significant only in 3rd trimester compared to 1st trimester. Such a difference in our results from previous report could be because of the use of cellular fractions instead of whole blood for mtDNA analysis. Still, our results are partly in agreement that the late stages of pregnancy (3rd trimester) show significantly reduced mtDNA content in the cellular fraction of maternal blood (almost half) compared to early gestation or non-pregnant conditions. We observed larger mean differences in these comparisons during effect size analysis, which suggests that the difference in cellular mtDNA levels in different trimesters has practical significance.

One of the potential reasons of such reduction in the mtDNA content might be because of infrequent feto-maternal hemorrhage (FMH) during late stages of physiological pregnancy during which, passage of fetal blood into maternal circulation through placenta may cause destruction of blood cells [13, 17]. Similarly, women with non-complicated pregnancy have relatively lower counts of platelets compared to non-pregnant women, and lowest at the time of delivery, which could be linked to reduced mtDNA content during pregnancy [18]. However, significant reduction in platelet counts with moderate-to-severe thrombocytopenia has been reported in pregnancies complicated with hypertensive disorders, such as preeclampsia [19]. Although we did not measure feto-maternal hemorrhage during pregnancy and excluded the pregnant women with hypertensive disorders, as discussed earlier; we retrospectively analyzed the blood profiling of pregnant group from different trimesters to understand the changes during pregnancy. While most hematological parameters in different trimesters (hemoglobin hematocrit, total leukocyte count, lymphocytes, eosinophils, and neutrophils) did not show any significant variations from 1st trimester, interestingly lower level of platelet in 3rd trimester was observed in our study cohort and it was significantly low compared to 1st trimester (Table 1). Although we did not have hematological data for the non-pregnant control group for comparison to pregnancy group, yet all these parameters are well within their normal range, including platelet counts during pregnancy according to the standard international reference values [20, 21].

Platelet count may significantly influence the mtDNA content measurement in the whole blood; and cell sorting could provide a better idea of changes in mtDNA content through a relative proportion of cell mixtures (i.e., leucocytes, lymphocytes, and platelets) [22]. Since the amount of sample was limiting in our pregnancy cohort and due to lack of hematological analysis in non-pregnant control samples, measurement of mtDNA content in different cell types could not be possible. However, to partly address the influence of platelet counts on mtDNA content, we reanalyzed the data and normalized the cellular mtDNA copy number values to the platelet counts. We found that there was no significant difference in mtDNA copy number among different trimesters upon normalizing with their respective platelet count, including the difference between 3rd vs 1st trimester (Supplementary Fig. 1). It shows that the observed significant difference of reduced cellular mtDNA content in 3rd trimester could be due to reduced platelet counts. However, it is important to mention that platelet activation upon stress conditions is also known to release mitochondria in circulation [23], which could cause reduced mitochondrial numbers in the platelet without altering the overall platelet numbers. In addition, late stages of pregnancy are associated with platelet activation and/or increased clearance [24]**,** which may also attribute to reduce mitochondrial content within platelets and also have effects on mitochondrial functionality. Thus, normalizing with platelet count alone may not address the reduced mtDNA content in blood. Therefore, it is difficult to link mtDNA and platelet count directly from our results without investigating these possibilities, which requires in-depth analysis of platelet mitochondrial biology and relative contribution from other cell types of blood during normal pregnancy. Comprehensive analysis of different cell types for mitochondrial functions/mtDNA content remains a limitation of the present study, which may have physiological relevance during pregnancy.

Conversely, in our study we found that plasma mtDNA was significantly higher in 3rd trimester compared to non-pregnant but did not differ significantly within different trimesters. Previously, in a study on absolute quantification of nuclear and mtDNA in serum during normal pregnancy, Cushen et al. reported a higher circulatory level of nuclear DNA as well as mtDNA during late pregnancy weeks (3rd trimester) compared to early or postpartum period [15]. In the same study, they reported that upon normalization to serum nuclear DNA, mtDNA remains higher in late pregnancy and postpartum compared to non-pregnant, with no difference among different gestational weeks. Although our results are based on relative quantification of mtDNA normalized to nuclear DNA content in plasma, it agrees with the previous report and suggests increased circulatory mtDNA content in 3rd trimester compared to the non-pregnant condition. In addition, effect size analysis also showed that the observed significant difference in plasma mtDNA content was attributed to larger mean differences of different trimesters vs non-pregnant than comparison within trimesters. While the origin of nuclear and mtDNA in circulation during pregnancy is not clear; it could result from leakage from cells during apoptosis/necrosis in pregnancy or as damage associated molecular patterns (DAMPs), which may initiate stress response including pro-inflammatory signaling [25–27].

It is known that, in normal pregnancy, various physiological changes occur that may contribute to maternal adaptation during pregnancy related changes. For example, development of placenta depends on implantation and invasion of the maternal decidua by the placental trophoblast cells [28]. In normal pregnancy, apoptosis of these trophoblast cells increases with advancing gestation and placental growth, leading to an increase in ruptured membrane particles in the maternal circulation from second trimester onwards [29]. Since a variety of stimuli may trigger placental apoptosis, including oxidative stress and hypoxia, a much higher level of placental apoptosis has been observed during pregnancy-related complications [29, 30]. In addition, leakage of mtDNA is an escape mechanism resulting from mitochondrial degradation during oxidative stress-induced apoptosis [31]. Such release of mtDNA could be mediated by Bax/Bak activation or voltage dependent anionic (VDAC) protein oligomerization and contribute to inflammatory responses [32, 33]. Thus, an increase in cell-free mtDNA as observed in our study could result from the presence of apoptotic bodies and/or leakage of mtDNA in circulation showing pregnancy related stress. In addition, recent reports also suggest that a large proportion of cell free DNA is contributed through extracellular vesicles [34], which are important to intracellular communication, adaptation to physiological changes, and feto-maternal vascular exchange during pregnancy [35]. With pregnancy complications, previous studies using whole blood or serum reported an elevated level of mtDNA content during PE condition [13, 36, 37]. However, in a recent study by Cushen et al. the opposite pattern for circulatory mtDNA in PE was also reported [26]. They suggested that most cell-free mtDNA is encapsulated in vesicles; leaving lower level of free mtDNA in plasma of PE compared to age-matched normal pregnant control [26]. Since the DNA isolation from plasma involves a lysis step, which allows the release of genetic material from exosomes/vesicles/apoptotic bodies (if any); our analysis in plasma represented the total mtDNA change in circulation during normal pregnancy. Similarly, an inverse correlation was also observed between cellular and circulatory mtDNA content in our study, showing that these two parameters might be inter-linked to each other. Whether, the decrease in mtDNA in cellular compartment and rise in plasma mtDNA content results from a combination of either increased apoptosis or mtDNA leakage or packaging of mtDNA in exosomes, especially during 3rd trimester needs further investigation. Thus, it would be interesting to further investigate the extent of apoptosis, level of apoptotic bodies or exosomes in plasma to understand mtDNA dynamics as suggested earlier [26].

As mentioned earlier, that reduced cellular mtDNA or increased circulatory mtDNA could be associated with damage response of physiological changes in advancing gestational period; we also investigated the oxidative status by measuring plasma H_2_O_2_ levels. A significantly higher H_2_O_2_ content across all trimesters was observed, which was aligned to the pattern of increased plasma mtDNA or reduced cellular mtDNA content compared to the non-pregnant group. However, within trimesters, elevated plasma H_2_O_2_ was only significant in 3rd trimester compared to 1st trimester and more aligned to the opposing pattern of reduced cellular mtDNA; which was also significantly reduced only in 3rd trimester compared to 1st trimester. These significant differences in 3rd trimester for plasma H_2_O_2_ or cellular mtDNA levels from 1st trimester were because of larger mean differences in these groups as analyzed by effect size (Table 2). It is known that systemic inflammatory response during normal pregnancy may cause oxidative stress and is exacerbated in pregnancy complications such as PE [38]. Physiological levels of ROS in normal pregnancy are involved in the activation of genes for oxygen sensing, differentiation, and proliferation, as well as to activate the host defense system [38]. In addition, increased ROS levels during late pregnancy are associated with placental membrane disruption and onset of labor, and shown to increase serum lipid oxidation during active labor [39, 40]. Since most of the pregnant participants in our study had term delivery (≥ 37 weeks), the observed increase in plasma H_2_O_2_ levels in 3rd trimester (32.8 weeks±1.30) shows an enhanced ROS signaling before the onset of term labor. Although, we did not investigate the compensatory increase in antioxidant defense system such as glutathione synthase, vitamin A, E and beta carotene, which are compromised in pregnancy-related complications [41], and could have provided a better idea of ROS regulation during normal pregnancy. While we observed a negative correlation between cellular mtDNA vs plasma H_2_O_2_ levels; plasma mtDNA and H_2_O_2_ levels were positively correlated with each other (Table 3). The correlation results show a link between reduced cellular mtDNA and/or increased plasma mtDNA levels with enhance ROS signaling during pregnancy, and might contribute to maternal adaptation to pregnancy related changes.

Small sample size is one of the potential limitations of our study, and we recognize these findings need to be replicated in a larger pregnancy cohort in a longitudinal study for further validation. In addition, cellular sorting of blood constituents could have provided a direct link of reduced cellular mtDNA content with specific cell type proportions in blood, which remains to be investigated. The source of circulating cell-free mtDNA present in the blood remains unclear from our study, which might have clinical implications and apply to future studies.

## Conclusions

Overall, our study clearly highlights that mtDNA content in cellular and cell-free fractions of blood shows opposing trend compared to non-pregnant condition. While the cellular mtDNA declines, the rise in circulatory H_2_O_2_ levels could be linked to increased cellular turnover and ROS signaling during late gestational weeks. Such pattern of mtDNA and circulating ROS changes could provide a basis for further investigating their absolute levels to set up a reference range for healthy pregnancy and could be extended to identify their prognostic/diagnostic potential for pregnancy-related complications. However, it warrants prior validation in different cohorts and correlation with age, sex of the fetus, ethnicity, and in different populations.

## Supplementary Information

**Additional file 1: Supplementary Figure 1.** Normalized cellular mtDNA content with platelet counts during pregnancy.

## Data Availability

The datasets used and/or analyzed during the current study are available from the corresponding author on reasonable request.

## References

[1] Spinelli JB, Haigis MC (2018). The multifaceted contributions of mitochondria to cellular metabolism. Nat Cell Biol.

[2] Perez M, Robbins ME, Revhaug C, Saugstad OD (2019). Oxygen radical disease in the newborn, revisited: oxidative stress and disease in the newborn period. Free Radic Biol Med.

[3] Murphy VE, Smith R, Giles WB, Clifton VL (2006). Endocrine regulation of human fetal growth: the role of the mother, placenta, and fetus. Endocr Rev.

[4] De Marco CS, Caniggia I (2002). Mechanisms of oxygen sensing in human Trophoblast cells. Placenta.

[5] Martinez F, Uribe A, Espinosa-Garcia MT, Flores-Herrera O, Garcia-Perez C, Milan R (2002). Calcium modulates the ATP and ADP hydrolysis in human placental mitochondria. Int J Biochem Cell Biol.

[6] Tuckey RC (2005). Progesterone synthesis by the human placenta. Placenta.

[7] Morén C, Hernández S, Guitart-Mampel M, Garrabou G (2014). Mitochondrial toxicity in human pregnancy: an update on clinical and experimental approaches in the last 10 years. Int J Environ Res Public Health.

[8] Anderson S, Bankier AT, Barrell BG, de Bruijn MHL, Coulson AR, Drouin J, Eperon IC, Nierlich DP, Roe BA, Sanger F (1981). Sequence and organization of the human mitochondrial genome. Nature.

[9] Babayev E, Seli E (2015). Oocyte mitochondrial function and reproduction. Curr Opin Obstetr Gynecol.

[10] Murakoshi Y, Sueoka K, Takahashi K, Sato S, Sakurai T, Tajima H, Yoshimura Y (2013). Embryo developmental capability and pregnancy outcome are related to the mitochondrial DNA copy number and ooplasmic volume. J Assist Reprod Genet.

[11] Duhig K, Chappell LC, Shennan AH (2016). Oxidative stress in pregnancy and reproduction. Obstetr Med.

[12] Busnelli A, Lattuada D, Ferrari S, Reschini M, Colciaghi B, Somigliana E, Fedele L, Ferrazzi E (2018). Mitochondrial DNA copy number in peripheral blood in the first trimester of pregnancy and different preeclampsia clinical phenotypes development: a pilot study. Reprod Sci.

[13] Colleoni F, Lattuada D, Garretto A, Massari M, Mandò C, Somigliana E, Cetin I (2010). Maternal blood mitochondrial DNA content during normal and intrauterine growth restricted (IUGR) pregnancy. Am J Obstet Gynecol.

[14] Williams MA, Sanchez SE, Ananth CV, Hevner K, Qiu C, Enquobahrie DA (2013). Maternal blood mitochondrial DNA copy number and placental abruption risk: results from a preliminary study. Int J Mol Epidemiol Genet.

[15] Cushen SC, Sprouse ML, Blessing A, Sun J, Jarvis SS, Okada Y, Fu Q, Romero SA, Phillips NR, Goulopoulou S (2020). Cell-free mitochondrial DNA increases in maternal circulation during healthy pregnancy: a prospective, longitudinal study. Am J Phys Regul Integr Comp Phys.

[16] Schmittgen TD, Livak KJ (2008). Analyzing real-time PCR data by the comparative CT method. Nat Protoc.

[17] Beneventi F, Cavagnoli C, Locatelli E, Bariselli S, Simonetta M, Viarengo G, Perotti C, Spinillo A (2018). Mild-to-moderate foeto-maternal haemorrhage in the third trimester and at term of pregnancy: quantitative determination and clinical-diagnostic evaluation. Blood Transfus.

[18] Reese JA, Peck JD, Deschamps DR, McIntosh JJ, Knudtson EJ, Terrell DR, Vesely SK, George JN (2018). Platelet counts during pregnancy. N Engl J Med.

[19] Ciobanu AM, Colibaba S, Cimpoca B, Peltecu G, Panaitescu AM (2016). Thrombocytopenia in pregnancy. Maedica (Bucur).

[20] Abbassi-Ghanavati M, Greer LG, Cunningham FG (2009). Pregnancy and laboratory studies. Obstet Gynecol.

[21] Morton A. Hematological Normal Ranges in Pregnancy. The Global Library of Women's Medicine 2021. ISSN: 1756-2228. 10.3843/GLOWM.413403.

[22] Picard M (2021). Blood mitochondrial DNA copy number: what are we counting?. Mitochondrion.

[23] Garcia-Souza LF, Oliveira MF (2014). Mitochondria: biological roles in platelet physiology and pathology. Int J Biochem Cell Biol.

[24] Shehata N, Burrows R, Kelton JG (1999). Gestational thrombocytopenia. Clin Obstet Gynecol.

[25] Collins LV, Hajizadeh S, Holme E, Jonsson I-M, Tarkowski A (2004). Endogenously oxidized mitochondrial DNA induces in vivo and in vitro inflammatory responses. J Leukoc Biol.

[26] Cushen SC, Ricci CA, Bradshaw JL, Silzer T, Blessing A, Sun J, Zhou Z, Scroggins SM, Santillan MK, Santillan DA, et al. Reduced Maternal Circulating Cell-Free Mitochondrial DNA Is Associated With the Development of Preeclampsia. J Am Heart Assoc. 2022;11(2):e021726. 10.1161/JAHA.121.021726 PMC9238514 35014857

[27] West AP, Shadel GS (2017). Mitochondrial DNA in innate immune responses and inflammatory pathology. Nat Rev Immunol.

[28] Knöfler M, Haider S, Saleh L, Pollheimer J, Gamage TKJB, James J (2019). Human placenta and trophoblast development: key molecular mechanisms and model systems. Cell Mol Life Sci.

[29] Sharp AN, Heazell AEP, Crocker IP, Mor G (2010). Placental apoptosis in health and disease. Am J Reprod Immunol.

[30] Tjoa ML, Cindrova-Davies T, Spasic-Boskovic O, Bianchi DW, Burton GJ (2006). Trophoblastic oxidative stress and the release of cell-free Feto-placental DNA. Am J Pathol.

[31] Pérez-Treviño P, Velásquez M, García N (2020). Mechanisms of mitochondrial DNA escape and its relationship with different metabolic diseases. Biochim Biophys Acta (BBA) - Mol Basis Dis.

[32] Galluzzi L, Vitale I, Aaronson SA, Abrams JM, Adam D, Agostinis P, Alnemri ES, Altucci L, Amelio I, Andrews DW (2018). Molecular mechanisms of cell death: recommendations of the nomenclature committee on cell death 2018. Cell Death Different.

[33] Kim J, Gupta R, Blanco LP, Yang S, Shteinfer-Kuzmine A, Wang K, Zhu J, Yoon HE, Wang X, Kerkhofs M (2019). VDAC oligomers form mitochondrial pores to release mtDNA fragments and promote lupus-like disease. Science.

[34] Camussi G, Fernando MR, Jiang C, Krzyzanowski GD, Ryan WL (2017). New evidence that a large proportion of human blood plasma cell-free DNA is localized in exosomes. PLoS One.

[35] Konečná B, Tóthová Ľ, Repiská G (2019). Exosomes-associated DNA—new marker in pregnancy complications?. Int J Mol Sci.

[36] Marschalek J, Wohlrab P, Ott J, Wojta J, Speidl W, Klein KU, Kiss H, Pateisky P, Zeisler H, Kuessel L (2018). Maternal serum mitochondrial DNA (mtDNA) levels are elevated in preeclampsia – a matched case-control study. Preg Hypertens.

[37] Qiu C, Hevner K, Enquobahrie DA, Williams MA (2012). A case-control study of maternal blood mitochondrial DNA copy number and preeclampsia risk. Int J Mol Epidemiol Genet.

[38] Chiarello DI, Abad C, Rojas D, Toledo F, Vázquez CM, Mate A, Sobrevia L, Marín R (2020). Oxidative stress: Normal pregnancy versus preeclampsia. Biochim Biophys Acta (BBA) - Mol Basis Dis.

[39] Agarwal A, Gupta S, Sharma RK. Role of oxidative stress in female reproduction. Reprod Biol Endocrinol. 2005;3:28. 10.1186/1477-7827-3-28 PMC1215514 16018814

[40] Fainaru O, Almog B, Pinchuk I, Kupferminc MJ, Lichtenberg D, Many A (2002). Active labour is associated with increased oxidisibility of serum lipids ex vivo. BJOG.

[41] Simsek M, Naziroglu M, Simsek H, Cay M, Aksakal M, Kumru S (1998). Blood plasma levels of lipoperoxides, glutathione peroxidase, beta carotene, vitamin a and E in women with habitual abortion. Cell Biochem Funct.

