# Supplementary material for: Analysis of cellular and cell free mitochondrial DNA content and reactive oxygen species levels in maternal blood during normal pregnancy: a pilot study
Source: BMC Pregnancy Childbirth. 2022 Nov 16;22:845. doi: 10.1186/s12884-022-05156-2 (PMC9670558; doi:10.1186/s12884-022-05156-2)
Supplement: Supplementary file 1 — Additional file 1: Supplementary Figure 1. Normalized cellular mtDNA content with platelet counts during pregnancy. [file 12884_2022_5156_MOESM1_ESM.docx]

**
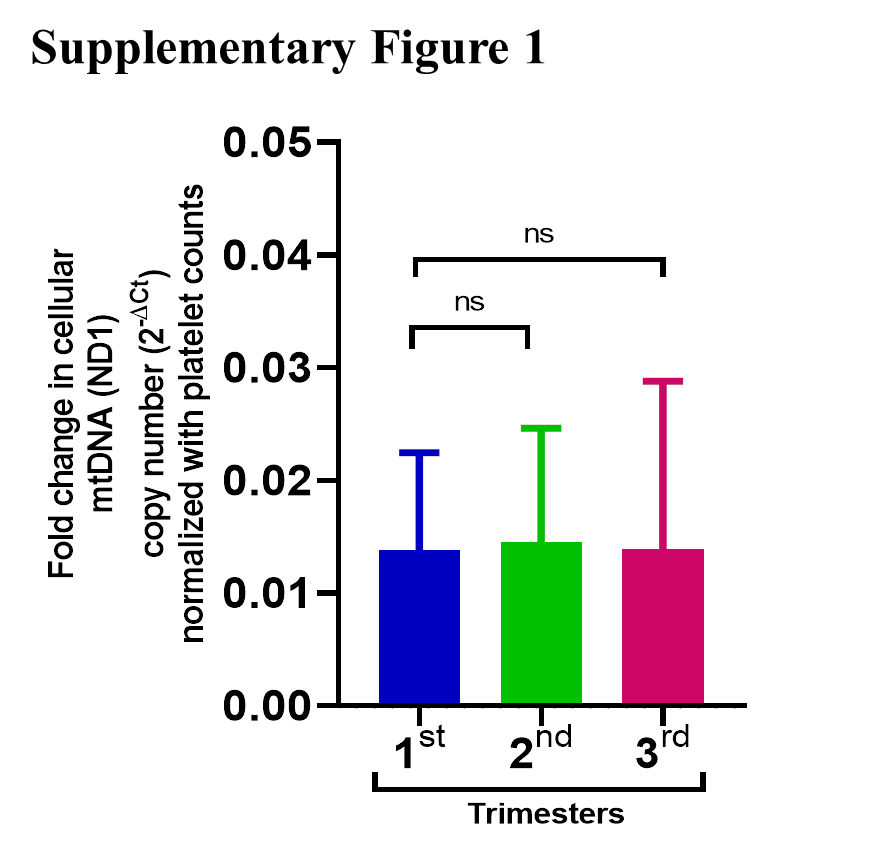
**

**Supplementary Figure 1: Normalized cellular mtDNA content with platelet counts during pregnancy:** Relative mtDNA copy number of different trimesters (2^-ΔCt^ values) was normalized with their respective plate counts and presented as fold change relative to 1^st^ trimester. Significance within different trimesters was calculated by Kruskal–Wallis H-test and post hoc analysis by Bonferroni Correction. ns: non-significant.
